# Comparative Transcriptome Analysis of the Accumulation of Anthocyanins Revealed the Underlying Metabolic and Molecular Mechanisms of Purple Pod Coloration in Okra (*Abelmoschus esculentus* L.)

**DOI:** 10.3390/foods10092180

**Published:** 2021-09-14

**Authors:** Yanjie Zhang, Tianjiao Zhang, Qing Zhao, Xiaodong Xie, Yan Li, Qiyan Chen, Fang Cheng, Jianwei Tian, Huihui Gu, Jinyong Huang

**Affiliations:** 1 School of Agricultural Sciences, Zhengzhou University, Kexue Avenue 100, Zhengzhou 450001, China; zhangyanjie@zzu.edu.cn (Y.Z.); zhangtianjiaosun@163.com (T.Z.); zhaoqing20200620@163.com (Q.Z.); qiyanchenn@gmail.com (Q.C.); cf199669@163.com (F.C.); tianjianwei0412@163.com (J.T.); hhgu@zzu.edu.cn (H.G.); 2 China Tobacco Gene Research Center, Zhengzhou Tobacco Research Institute of CNTC, Fengyang Avenue, Zhengzhou 450001, China; xiexd@cqu.edu.cn; 3 The Center of Advanced Analysis and Gene Sequencing, Zhengzhou University, Kexue Avenue 100, Zhengzhou 450001, China

**Keywords:** okra, pod, anthocyanin, accumulation, transcriptome, transcriptional regulation

## Abstract

Color is an essential agronomic trait and the consumption of high anthocyanin containing vegetables in daily diet does provide benefits to human health, but the mechanisms on anthocyanin accumulation in tender pods of okra (*Abelmoschus esculentus* L.) were totally unknown. In this study, a wide characterization and quantitation of anthocyanins and flavonols in tender pods of 15 okra varieties were performed by UHPLC-Q-Orbitrap HRMS for the first time. Two major anthocyanins (delphinidin 3-*O*-sambubioside and cyanidin 3-*O*-sambubioside) and six kinds of flavonol glycosides (most are quercetin-based) were identified and quantified. The coloration of the purple okra pod mainly arises from the accumulation of both delphinidin 3-*O*-sambubioside and cyanidin 3-*O*-sambubioside in most of purple varieties (Hong Yu, Bowling Red and Burgundy), except Jing Orange. The significant differences in the compositions and contents of anthocyanins are responsible for the pod color ranging from brick-red to purplish-red among the various okra cultivars. Furthermore, four representative okra cultivars exhibiting obvious differences in anthocyanin accumulation were further analyzed with transcriptome and more than 4000 conserved differentially expressed genes were identified across the three compared groups (B vs. BR, B vs. HY and B vs. JO). Based on the comprehensive analysis of transcriptomic data, it was indicated that MBW complex consisting of AeMYB114, AeTT8, and AeTTG1 and other transcriptional factors coordinately regulate the accumulation of anthocyanins via the transcriptional regulation of structural genes. Moreover, four independent working models explaining the diversities of anthocyanin pigmentation in okra pods were also proposed. Altogether, these results improved our understanding on anthocyanin accumulation in okra pods, and provided strong supports for the development of okra pod as a functional food in the future.

## 1. Introduction

As natural water-soluble pigments, anthocyanins are widely distributed in land plants and generate the characteristic red, purple, and blue colors in plant tissues and organs, including the leaves, stems, roots, flowers, seeds, and fruits [1]. Apart from serving as pollinators and seed dispersers, anthocyanins provide protection against abiotic and biotic stresses including UV radiation, cold, drought stress, and microbial agents [2,3,4]. Due to the high antioxidant activity, anthocyanins have health-promoting properties [5,6]. Growing evidences reveal that the regular consumption of anthocyanins can lower the risk of inflammation, cardiovascular diseases, age-related degenerative diseases (such as diabetes, atherosclerosis), and certain cancers [7,8,9,10].

The anthocyanin biosynthetic pathway which derives from flavonoid pathway has been extensively studied in Arabidopsis (*Arabidopsis thaliana*), grape (*Vitis vinifera*), petunia (*Petunia hybrida*), snapdragon (*Antirrhinum majus*), maize (*Zea mays*), blood orange (*Citrussinensis Osbeck* L.), and tomato (*Solanum lycopersicum*) [5,11,12,13]. Generally, the structural genes in the flavonoid pathway are highly conserved in plants. Flavonoid biosynthesis begins with the cleavage of phenylalanine catalyzed by phenylalanine ammonia-lyase (PAL), resulting in the generation of cinnamic acid. Subsequently, dihydrokaempferol is produced from cinnamic acid by a series of enzymes including cinnamate 4-hydroxylase (C4H), 4-coumaroyl: CoA-ligase (4CL), chalcone synthase (CHS), chalcone isomerase (CHI), and flavanone 3-hydroxylase (F3H). The dihydrokaempferol can be further converted to dihydroquercetin and dihydromyricetin by flavanone 3′-hydroxylase (F3′H) and flavanone 3′,5′-hydroxylase (F3′5′H), respectively. Usually, the enzyme F3′H hydroxylate the B-ring of dihydrokaempferol to form cyanidin while F3′5′H hydroxylate the B-ring to produce delphinidin eventually. Accordingly, hydroxylation degree of the B-ring confers significant changes in the color range of flavonoids, including anthocyanidins [1]. Colorful anthocyanidins are synthesized through the subsequential reaction catalyzed by dihydroflavonol 4-reductase (DFR) and anthocyanidin synthase (ANS) from the dihydroflavonols. Meanwhile, flavonols can be synthesized from dihydroflavonols by flavonol synthase (FLS). Finally, anthocyanidins and flavonol aglycones are further glycosylated and acetylated to generate diverse stable products which are accumulated widely in plant cells [14].

Anthocyanin biosynthesis is mainly regulated at transcription level [15]. The expression of structural genes is directly triggered by a transcriptional complex, consisting of R2R3-MYB, basic-helix-loop-helix (bHLH), and WD-repeat (WDR) proteins (MBW complex) [1,15,16]. Besides, several R2R3-MYBs including AtMYB11, AtMYB12 and AtMYB111, with a high degree of functional similarity, control flavonol biosynthesis specifically in tissues of aerial part [17]. On the contrary, partial members of R3-MYB and R2R3-MYB proteins act as repressors in the regulation of anthocyanin and flavonol [16,18]. For instance, AtMYB4, a R2R3-MYB repressor, negatively regulates the transcription of the C4H in response to UV-B irradiation [19]. Besides, AtMYBL2, a R3-MYB repressor, suppressed the expression of DFR and TT8, thereby reducing the metabolic flux towards anthocyanins in Arabidopsis; CPC, another R3-MYB repressor, act as a competitive inhibitor by binding with bHLH proteins in both A. thaliana and petunia, resulting in the compromising formation of a functional MBW complex [20,21,22]. Furthermore, the anthocyanin biosynthetic genes can also be regulated directly or indirectly by other TFs, such as by WRKY, bZIP, NAC, and MADS-box transcriptional factors [23,24,25].

Okra (*Abelmoschus esculentus* L.), a member of the mallow family, is an important vegetable crop and widely grown, especially in tropical to subtropical regions [26]. Okra is considered as a valuable crop due to the multiple functions in traditional and modern medicines. Okra pods, rich in glycoproteins, dietary fiber, vitamins, flavonoids, and mineral elements, are beneficial to the digestive and immune systems and are used as food additives because of their antigastric acid, antifatigue, antioxidation, and anti-inflammation properties [27]. Recently, the purple or red okra cultivars which show abundant accumulation of anthocyanins have received much attention from consumers due to the appealing color and health-promoting ingredients. However, the studies on the anthocyanin pigmentation in okra pods are very limited and the underlying mechanisms in purple okra also remain unclear.

In this study, 15 okra cultivars which show a wide range of color and significant differences in anthocyanin accumulation in pods were selected as materials. To investigate the purple pigments produced in okra, ultrahigh performance liquid chromatography coupled with quadrupole Orbitrap high-resolution mass spectrometry (UHPLC-Q-Orbitrap HRMS) were used to identify and quantify the anthocyanins produced in the okra pods. Compared to the green cultivars, two major anthocyanins were identified from pods of the purple okra cultivars. In addition, six major flavonol glycosides were also identified and quantified in all the okra cultivars. Furthermore, three purple cultivars and one green cultivar with a similar pod shape, were selected for RNA-Seq and comparative transcriptome analysis to explore the underlying mechanisms for anthocyanin accumulation in okra. In summary, it is the first time that anthocyanin accumulation was systemically studied and these results will certainly fill the gaps in the coloration of okra pod.

## 2. Materials and Methods

### 2.1. Plant Materials

**Figure 1:**
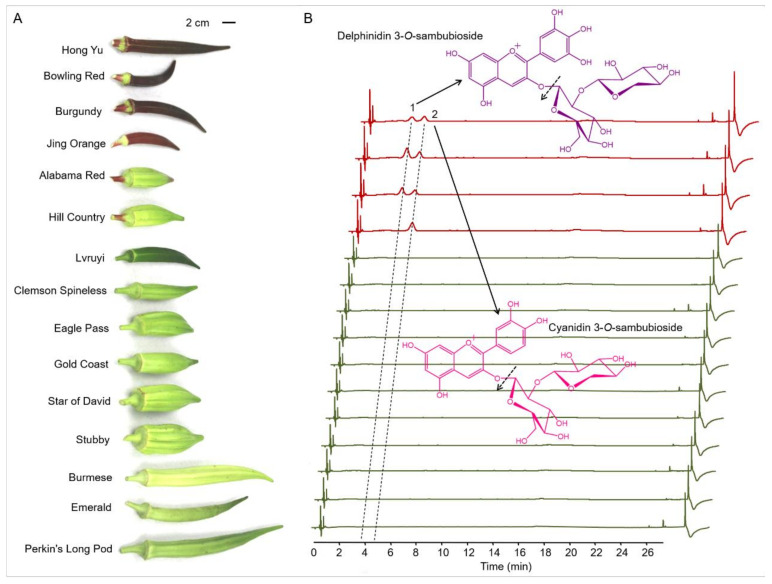
Anthocyanins identified in the pods from 15 okra cultivars using UHPLC-Q-Orbitrap HRMS (Ultra-High Performance Liquid Chromatography coupled with quadrupole Orbitrap high-resolution mass spectrometry). (**A**) Okra pods display multifarious colors and shapes in various cultivars. (**B**) UHPLC profiles of the anthocyanins extracted from the tender pods of the 15 okra cultivars. Major cleavage sites of delphinidin 3-*O*-sambubioside and cyanidin 3-*O*-sambubioside conformed by mass spectrometry were indicated by dashed arrow.

The seeds of the 13 heirloom cultivars of okra (*Abelmoschus esculentus* L.) were obtained from Baker Creek Heirloom Seed Company (Missouri, USA). Two representative commercial okra cultivars (Hong Yu and Lvruyi) were bought from local seed company in China (Zhengzhou, China). Four purple cultivars (Hong Yu, Bowling Red, Burgundy, and Jing Orange) show apparent accumulation of purple pigments in pod skins and eleven cultivars (Alabama Red, Hill Country, Lvruyi, Clemson Spineless, Eagle Pass, Gold Coast, Star of David, Stubby, Burmese, Emerald and Perkin’s Long Pod) show green pods with different degrees of intensities (Figure 1). All the okra cultivars were grown in a uniform environment in a test plot at Zhengzhou, in the months of May–July, 2019. Possible drought-related stress was eliminated by frequently watering. The tender pods were harvested on the 10th day after anthesis and the skins were carefully separated and immediately flash-frozen in liquid nitrogen.

### 2.2. Chemicals and Reagents

Ultrapure grade water was prepared by a Milli-Q purification system. Methanol, acetonitrile, and formic acid were bought from Merck (Merck KGaA, Darmstadt, Germany). Anthocyanins (cyanidin 3-*O*-sambubioside and delphinidin 3-*O*-sambubioside) for the external standards were obtained from Phytolab (PhytoLab GmbH & Co. KG, Dutendorfer Str. 5-7, 91487 Vestenbergsgreuth, Germany). Quercetin 3-*O*-glucoside, kaempferol 3-*O*-glucoside, myricetin 3-*O*-glucoside and the other reagents were bought from Sigma-Aldrich.

### 2.3. RNA Preparation and RNA-Seq

Total RNAs were extracted from the pod skins of three purple okra cultivars (Hong Yu, Bowling Red, and Jing Orange) and one green cultivar (Burmese) with TRIzol reagent according to the manufacturer’s instructions. DNA contamination was eliminated by DNase I treatment. RNA integrality was analyzed by RNase free agarose gel electrophoresis. The quality and quantity of the RNA samples were examined with Bioanalyzer 2100 system (Agilent Technologies, California, USA) and Nanodrop One spectrophotometer (Life Technologies).

The total RNAs extracted from pod skins of the four okra cultivars were used to construct the RNA-Seq library, respectively. Each okra cultivar was analyzed in three replicates. The construction of the cDNA libraries and RNA-seq were performed by Metware Biotechnology Co., Ltd. (https://www.metware.cn (PRJNA761494)) on the Illumina HiSeq 4500 platform.

### 2.4. De Novo Transcriptome Assembly and Annotation

Clean reads were obtained by removing the adaptor and reads of low quality from the original data generated by high-throughput sequencing. Next, the GC contents, Q20 and Q30, were calculated for quality control. Then, the clean reads were processed for de novo assembly using Trinity tool kit (version: v2.6.6). High quality reads obtained by the method of de novo assembly were called as transcripts and assembled into unigenes with Corset software. Finally, all unigenes were analyzed using Blast (with E-value < 1 × 10^−5^) in protein databases, such as NR (nonredundant protein database), Swiss-Prot (annotated protein sequence database), KEGG (Kyoto Encyclopedia of Genes and Genomes), KOG/COG (Clusters of Orthologous Groups of proteins/euKaryotic Ortholog Groups), GO (gene ontology), and Trembl, to obtain functional annotations.

### 2.5. Identification of Differentially Expressed Genes (DEGs)

Quantification of the gene expression levels was estimated by Fragments Per Kilobase of exon model per Million mapped fragments (FPKM). RSEM software was used in the quantification of gene expression levels, and the counts were then normalized to the FPKM values. The differentially expressed genes among the pod skins of four okra cultivars were identified by DESeq (ver. 1.22.2). A fold change (FC) value of ≥ 2 and a false discovery rate (FDR) of < 0.01 were used as criteria for selecting DEGs. A heat map was generated using R (ver. 3.5.1) software.

### 2.6. Extraction and UHPLC-Q-Orbitrap HRMS Analysis of Flavonoids

Pod skins of the 15 okra cultivars were first ground into fine powder with liquid nitrogen and then freeze-dried. Flavonoids were extracted according to the methods reported in previous studies with slight modification [26]. Briefly, 50 mg of lyophilized samples were leached by ultrasonic treatment for 30 min in 5 mL of CH_3_OH/H_2_O (75:25, *v*/*v*) containing 1% acetic acid, and then the suspension placed on ice overnight. After centrifugation at 13,000 rpm for 10 min, the supernatants were filtered through a 0.22 μm PTFE membrane for UHPLC-Q-Orbitrap HRMS analysis.

One microliter of the extracts was injected for the separation of anthocyanins and flavonols by a Vanquish Flex UHPLC system (Thermo Scientific, Waltham, MA USA) with a Waters XBridge C18 column (1.7 μm, 2.1 mm × 150 mm). The eluates were first analyzed with a variable-wavelength detector. Anthocyanins and flavonols were analyzed with UV detection at 535 nm and 360 nm, respectively. A binary mobile phase consisting of (LC/MS)-grade water (containing 0.1% formic acid; solvent A) and acetonitrile (solvent B) was applied for analysis. Gradient elution program was as follows: initial, 2% B; at 0.5 min, 2% B; at 2 min, 8% B; at 8 min, 15% B; at 16 min, 18% B; at 26 min, 95% B; at 28 min, 95% B; at 28.1 min, 95% B; at 30.10 min, 2% B. The flow rate was set to 0.35 mL min-1. Anthocyanin and flavonol quantification were expressed as their respective external standard equivalent.

A Q-Exactive mass spectrometer (Thermo Scientific) coupled to the UHPLC system was used for the identification of the target metabolites. Xcalibur 2.3 was used for data acquisition. The Q-Exactive mass spectrometer was executed according to the method described previously [27]. Metabolites were identified by comparing their retention time and mass spectra with those of the authentic compounds.

### 2.7. Statistical Analysis

Data from three biological replicates were used for the statistical analysis with SPSS, version 21.0 (SPSS Inc., Chicago, IL, USA). Data were presented as the mean ± standard deviation (SD). One-way analysis of variance (ANOVA) was followed by Duncan’s tests. The significance level was set to *p* < 0.05. Different letters indicated the results were statistically significant at *p* < 0.05.

## 3. Results

### 3.1. Identification and Quantitation of Anthocyanins in Pods of 15 Okra Cultivars

To explore the metabolic mechanisms of purple pod coloration, a total of 15 okra cultivars including four purple cultivars and 11 green cultivars were selected for study. Visual inspection of the 15 okra cultivars shows that four purple cultivars (Hong Yu, Bowling Red, Burgundy and Jing Orange) exhibit apparent accumulation of purple pigments compared with the other eleven cultivars exhibiting green pods. In addition, the intensities of the purple pigments accumulated in the pod skins of Hong Yu, Bowling Red and Burgundy are apparently stronger than those in Jing Orange (Figure 1A). As the purple pigments extracted from okra pods of all the purple cultivars are water-soluble, the coloration of the purple okra is speculated to arise from the accumulation of anthocyanins, not betacyanin or carotenoids.

To investigate the precise components of these eye-catching purple pigments, the total flavonoid extractions from the tender pods (10 d after anthesis) were analyzed carefully with the application of UHPLC-Q-Orbitrap HRMS. Due to the characteristic absorption peaks of anthocyanins in the UV−vis region, the wavelength for anthocyanin identification and quantification was set at 535 nm [28]. Through the UHPLC analysis of the total flavonoids extracted from the pod skins of the 15 okra cultivars with a detection wavelength at 535 nm, two major chromatogram peaks (marked with peak 1 and peak 2) of potential anthocyanins were detected in pod skins of Hong Yu, Bowling Red and Burgundy, while only one major peak (peak 2) was found in pod skins of Jing Orange (Figure 1B). We further identified and analyzed these two potential anthocyanins by comparing their UV−vis absorption spectra, retention time, accurate mass, and product ions to those of standards and previous studies [29,30]. The results showed peak 1 and peak 2 were identified to be delphinidin 3-*O*-sambubioside and cyanidin 3-*O*-sambubioside, respectively (Figure 1B and Table 1).

Total anthocyanin contents were further measured among the okra cultivars. Total contents of anthocyanins in the pod skins of Hong Yu, Bowling Red, Burgundy, and Jing Orange are 6.99, 11.05, 7.35, and 4.41 mg/g (dry weight), respectively (Table 1). On the contrary, no visible signals of anthocyanins were detected in pod skins of all the green okra cultivars. It is remarkable that the ratios of delphinidin 3-*O*-sambubioside to cyanidin 3-*O*-sambubioside among the four purple okra cultivars varied vastly depending on okra variety. In the pod skins of Hong Yu, the content of delphinidin 3-*O*-sambubioside is roughly equivalent to that of cyanidin 3-*O*-sambubioside, while the levels of delphinidin 3-*O*-sambubioside in both Bowling Red and Burgundy are apparently more than those of cyanidin 3-*O*-sambubioside. By contrast, there is no evident signal of delphinidin 3-*O*-sambubioside detected in the pod skin of Jing Orange. The structure difference between these two anthocyanin molecules lies in the hydroxylation degree of C3′ and C5′ of B ring [1]. As the hydroxylation of the B-ring confer significant increase in maximum absorption wavelength (bathochromic shift) and coloration changes of anthocyanidins, it is rational to attribute the brick-red coloration of the Jing Orange pod to the only accumulation of cyanidin 3-*O*-sambubioside (Figure 1A and Table 1). Likewise, the purplish-red coloration of the pods of Hong Yu, Bowling Red, and Burgundy should be caused by the concurrent accumulation of cyanidin 3-*O*-sambubioside and delphinidin 3-*O*-sambubioside (Figure 1 and Table 1). Additionally, all the anthocyanins identified in okra pods are glycosylated at C3 position in C-ring with glucose and subsequent xylose, suggesting a common modification pattern for anthocyanidin aglycones in various cultivars or germplasm resources of okra (Figure 1 and Table 1). Altogether, these results indicate that the diverse kinds and contents of anthocyanins produced in the pod skins might result from the specific genotypes of the okra cultivars or germplasm resources.

### 3.2. Identification and Quantitation of Flavonols in Various Okra Pods

**Figure 2:**
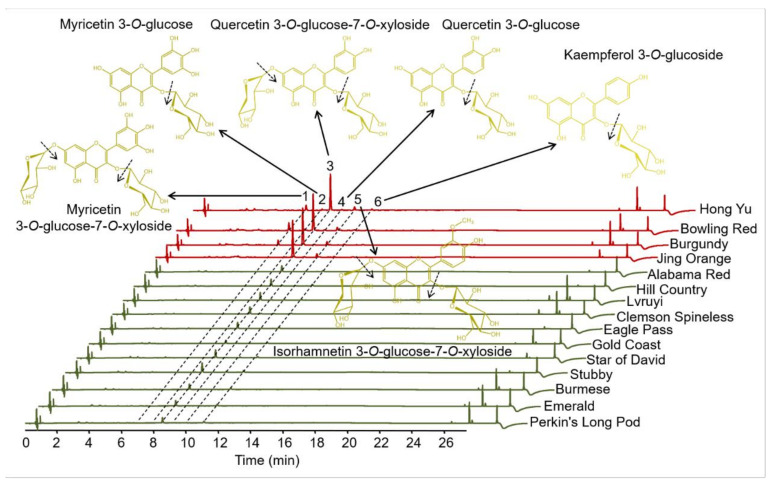
UHPLC-Q-Orbitrap HRMS analysis of flavonol glycosides in okra pods of various cultivars. Major cleavage sites of the flavonol glycosides confirmed by mass spectrometry are indicated by dashed arrows, respectively.

Similar with anthocyanins, flavonols are an important class of bioactive compounds widely reported as providing health promoting benefits, primarily through the properties of antioxidant or anti-inflammatory. Among flavonoids, flavonol represent one of the most important class in terms of concentration such as in onions and white grapes [31]. Additionally, flavonols can stabilize the color of anthocyanins through co-pigmentation, and the total amount of flavonols varies extensively among various varieties of many plant species [32]. To further reveal the mechanisms underlying pod coloration, the flavonols in pod skins were carefully identified and quantified in 15 cultivars. Due to the B-ring in the molecular structure, typical UV-vis spectra of flavonoids have Band I with a maximum in the 300–550 nm range and the A-ring contributes to Band II with a maximum in the 240–285 nm range [33]. Based on this, we measured the apparent absorption peaks of flavonoids at the wavelength of 360 nm. Among the 15 okra cultivars, there are six obvious chromatographic peaks detected by the UHPLC analysis (Figure 2 and Table 2). With the results of mass spectrometer, these flavonoids were further identified by the comparison of their accurate mass, retention times, and UV−vis spectra to standards and the literature [34]. All the six chromatographic peaks exhibited in Figure 2 were identified to be flavonol glycosides: myricetin 3-*O*-glucose-7-*O*-xyloside, myricetin 3-*O*-glucoside, quercetin 3-*O*-glucose-7-*O*-xyloside, quercetin 3-*O*-glucoside, isorhamnetin 3-*O*-glucose-7-*O*-xyloside, and kaempferol 3-*O*-glucoside (Figure 2 and Table 2).

The contents of the major flavonol in the 15 okra cultivars were calculated (Table 3). The total contents of flavonols in the pod skins of the four purple cultivars (4.85~5.59 mg/g dry weight) were obviously higher than those of the eleven anthocyaninless cultivars (0.37~1.35 mg/g dry weight). Among all the 15 okra cultivars, quercetin 3-*O*-glucose-7-*O*-xyloside occupies the highest proportion in the total flavonoids (68.12~89.57%). In the purple okra cultivars, the proportion of myricetin 3-*O*-glucose-7-*O*-xyloside (10.02~14.63%) were ranked the second, followed by that of quercetin 3-*O*-glucoside (5.85~7.01%), except for Jing Orange. In the pod skin of Jing Orange, quercetin 3-*O*-glucoside and isorhamnetin 3-*O*-glucose-7-*O*-xyloside were measured to be the second and the third most abundant flavonols, respectively. By contrast, the second highest proportion of flavonols in the other eleven anthocyaninless cultivars was isorhamnetin 3-*O*-glucose-7-O-xyloside (10.53~29.63%), followed by quercetin 3-*O*-glucoside (1.85~7.56%) with the exclusion of Burmese for the undetectable signal of quercetin 3-*O*-glucoside. In the purple okra varieties, it was evident that none of the myricetin-based flavonol glycosides were detected in the pod skin of Jing Orange. The absence of myricetin-based flavonol glycosides and delphinidin-based anthocyanins was the result of the short of precursor dihydromyricetin, suggesting the lack of F3′5′H activity in Jing Orange. Likewise, myricetin-based flavonol glycosides were absent in all the pod skins of anthocyaninless cultivars, as well as Jing Orange.

In many plants, an increase in flavonol accumulation was usually concurrent with the enhanced production of anthocyanins, because the augmented metabolic flux toward anthocyanin accumulation in flavonoid pathway can supply more intermediates, which finally resulted in the enhanced accumulation of flavonol glycosides in purple cultivars [26,27]. Accordingly, it is easy to speculate that the accumulation of a small amount of isorhamnetin 3-*O*-glucose-7-*O*-xyloside in okra pods arise from the derivatization of quercetin 3-*O*-glucose-7-*O*-xyloside catalyzed by endogenous flavonoid 3′-*O*-methyltransferase [35]. Except myricetin-based flavonol glycosides, all the pod skins of anthocyaninless cultivars lacked kaempferol 3-*O*-glucoside, and quercetin-based flavonols represent the majority. In the end, it is worth emphasizing that no evident signals of flavonol aglycones were detected in okra pods. Altogether, some conclusions about flavonol accumulation might be summarized: (1) overwhelming majority of flavonols in okra pods exist in the glycoside formation, suggesting that flavonol aglycones are unstable and easily modified by the endogenous flavonoid glycosyltransferases with high activities; (2) quercetin-based flavonols is accumulated by priority in flavonoid pathway.

### 3.3. RNA-Seq, De Novo Assembly, and Functional Annotation

To investigate the molecular mechanisms underlying the reinforced accumulation of anthocyanins in okra pods, twelve cDNA libraries constructed by the total RNAs from the skins of green (Burmese: B) and purple cultivars (Hong Yu: HY; Bowling Red: BR; and Jing Orange: JO) were characterized by the Illumina RNA-seq technology for further transcriptome analysis in three biological replicates. A summary of RNA-Seq data is shown in Table S1. After removing the adaptor sequences, high-quality and clean reads were obtained by removing low-quality and ambiguous reads in each library. The Q30 content of the 12 cDNA libraries was more than 87%, and the GC content was about 45%. The Trinity program was used to assemble all clean reads, and a total of 404,809 unigenes were detected by Bowtie2 (Table S2) and DESeq software with the length distribution of these unigenes was shown in Figure S1. Principal component analysis (PCA) of the samples based on the number of fragments per kilobase of exon per million fragments mapped values (FPKM) showed that all the biological replicates clustered together, indicating the high reliability of our sequencing data (Figure S2). Subsequently, the functional annotations were analyzed. Among them, 242,070 (59.80%), 347,717 (85.90%), 249,433 (61.62%), 346,635 (85.63%), 200,503 (49.53%), 296,818 (73.32%), and 227,299 (56.15%) unigenes were annotated to the KEGG, NR, SwissProt, Trembl, KOG, GO and Pfam databases, respectively (Table S3). The transcriptome assembly results revealed that the RNA-Seq datasets were reliable for further study.

### 3.4. Differentially Expressed Genes between Pod Skins of the Green and Purple Okra Cultivars

**Figure 3:**
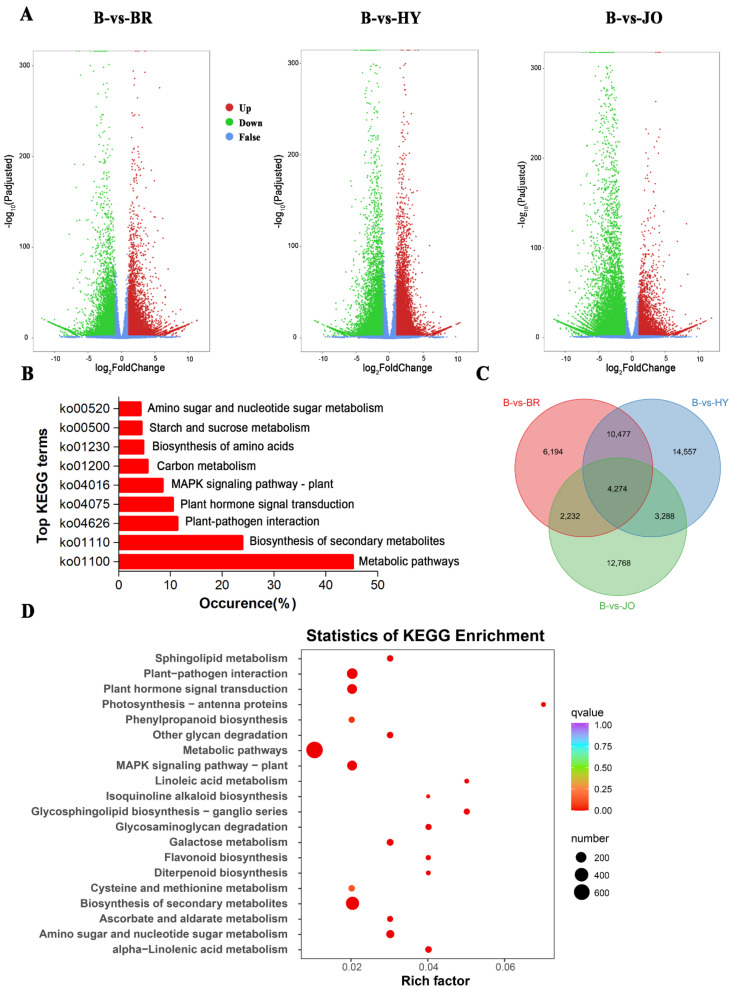
Differentially expressed unigenes between pod skins of the green and purple okra (B vs. BR, B vs. HY, and B vs. JO). (**A**) Volcano plots displaying the up−regulated, down−regulated, and no−regulated genes of the three compared groups. (**B**) Top KEGG terms contributed by all the differentially expressed unigenes. (**C**) Venn diagram showing the shared and the unique differentially expressed unigenes across the three compared groups of okra pods. (**D**) The top 20 of KEGG enrichment of the 4,274 differentially expressed unigenes shared by the three compared groups.

**Figure 4:**
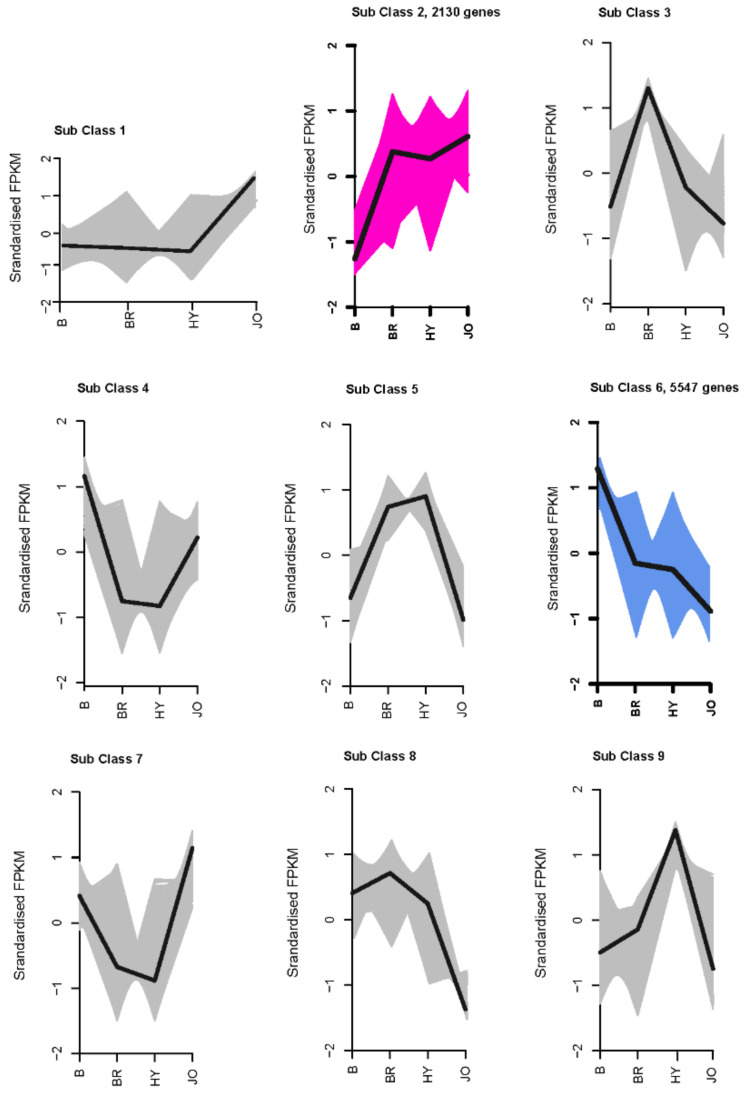
Expression patterns of all the differentially expressed genes in the pod skins of the three purple okra cultivars in comparison with the green cultivar.

The differentially expressed genes between the green and purple okra pod peels were selected according to the criterion of the log2|Fold Change| > 1 and FDR < 0.01. We identified 23,177, 32,596, and 22,562 differentially expressed unigenes for B vs. BR, B vs. HY and B vs. JO, respectively (Figure 3 and Table S4). Among differentially expressed unigenes, 12,307, 18,051 and 7381 were upregulated while 10,870, 14,545, and 15,181 were found be downregulated in B compared with BR, HY, or JO, respectively. And then, all differentially expressed unigenes between the green and purple okra pod peels (B vs. BR, B vs. HY and B vs. JO) were grouped in clusters according to their FPKM data using k-mean cluster analysis. In total, nine expression patterns of the DEGs were clustered. And the subclass 2, consisting of 2130 genes, showed significant up-regulation in all purple okra pod peels compared with the green cultivar (Figure 4 and Table S5). Remarkedly, the subclass 2 contains many structural genes of the flavonoid biosynthetic pathway, suggesting the fundamental roles of flavonoids, especially anthocyanins play in okra pod coloration. Conversely, 5547 genes in sub class 6 were negatively associated with the purple pod coloration as they were gradually down-regulated in purple okra cultivars (Figure 4 and Table S6). Interestingly, 2 MYB transcription factor unigenes (Cluster-2683.247477 and Cluster-2683.168435) in sub class 2 show a significant increase in expression levels, while 32 MYB unigenes from the sub class 6 displayed a decrease in expression levels in purple okra pods with the green okra cultivar as a control, suggesting their potential regulatory roles in purple okra pod coloration.

All the differentially expressed unigenes were assigned to KEGG enrichment analysis. The top enriched KEGG terms contributed by all differentially expressed unigenes were ko01100 (metabolic pathways), ko01110 (biosynthesis of secondary metabolites), ko04626 (plant-pathogen interaction), ko04016 (MAPK, mitogen-activated protein kinase, signaling pathway-plant), ko01200 (carbon metabolism), ko01230 (biosynthesis of amino acids), ko00520 (amino sugar and nucleotide sugar metabolism) and ko00500 (starch and sucrose metabolism) (Figure 3B). We then clustered all three lists of differentially expressed unigenes and obtained 4274 common differentially expressed unigenes totally across the three compared groups (Figure 3C and Table S7). These conserved differentially expressed unigenes were enriched for KEGG terms involved in metabolic pathways (ko01100), biosynthesis of secondary metabolites (ko01110), plant-pathogen interaction (ko04626), and MAPK signaling pathway—plant (ko04016). Besides, the pathways of the flavonoid biosynthesis and phenylpropanoid biosynthesis involved in anthocyanin and flavonoid accumulation were apparently enriched in the purple okra pods (Figure 3D). Interestingly, some differentially expressed unigenes were significantly enriched in photosynthesis-antenna proteins with the highest rich factor and the similar biological phenomenon were also reported in other species [26,27], implying that anthocyanins not only function as UV-protecting sunscreens but also regulate the energy absorbed by light harvesting proteins during photosynthesis in higher plants.

Concerning our research, differentially expressed unigenes involved in the phenylpropanoid biosynthesis (ko00940), flavonoid biosynthesis (ko00941) and anthocyanin biosynthesis pathway (ko00942) were further analyzed according to the KEGG database. There were 177, 263, and 195 differentially expressed unigenes involved in phenylpropanoid biosynthesis for B vs. BR, B vs. HY and B vs. JO, respectively. 76, 90 and 62 differentially expressed unigenes participated in flavonoid biosynthesis and 5, 27 and 17 differentially expressed unigenes enriched in anthocyanin biosynthesis pathway were identified across the three compared groups, respectively. To explore the molecular mechanisms underlying pod skin coloration, these genes were further analyzed.

### 3.5. Expression Analysis of Anthocyanin and Flavonol Biosynthetic Genes in Pod Skins of the Four Okra Cultivars

**Figure 5:**
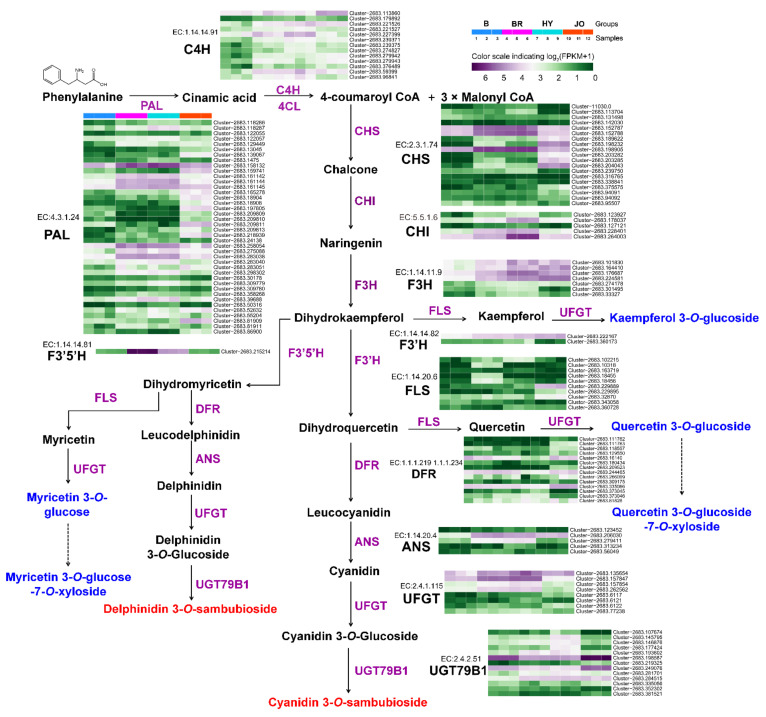
Transcript profiling of the anthocyanin and flavonol biosynthetic genes in pod skins of the four okra cultivars. phenylalanine ammonia lyase, PAL; cinnamate 4-hydroxylase, C4H; 4-coumarateCoA ligase, 4CL; chalcone synthase, CHS; chalcone isomerase, CHI; flavanone 3-hydroxylase, F3H; flavonol synthase, FLS; flavonoid 3′-hydroxylase, F3′H; flavonoid 3′,5′-hydroxylase, F3′5′H; dihydroflavonol, 4-reductase, DFR; anthocyanidin reductase, ANR; UDP-glycose: flavonoid gly-cosyltransferase, UFGT.

To further understand the coloration of okra pods, unigenes encoding proteins associated with anthocyanin biosynthesis were firstly identified (Figure 5). In comparison with the green cultivar Burmese, most of the anthocyanin biosynthetic genes were significantly up-regulated in pod skins of BR, HY and JO. Especially, EC:4.3.1.24 (phenylalanine ammonia lyase, PAL), EC:2.3.1.74 (chalcone synthase, CHS), and EC:1.14.11.9 (flavanone 3-hydroxylase, F3H) showed an enormous increase in gene expression levels. These up-regulated structural genes with high expression levels enhanced the metabolic flux towards anthocyanins and flavonols in the purple okra pods, compared with the green cultivar. Notably, EC:1.14.14.81 (flavonoid 3′,5′-hydroxylase, F3′5′H/CYP75A) was specifically expressed in both BR and HY cultivars with undetectable transcripts in the pod skins of both B and JO. The enhanced catalytic activity of F3′5′H resulted in the hydroxylation of B-ring at C5′ for the production of delphinidin-based anthocyanins and myricetin-based flavonol glycosides in the pods of both BR and HY cultivars, while these metabolites were absent in JO and B for the extreme low transcript abundance of F3′5′H. Similarly, it is easy to understand the lack of myricetin-based flavonol glycosides in the other 10 green okra cultivars.

In plant, glycosylation is a widespread modification pattern for many secondary metabolites, and anthocyanidins and other flavonoid aglycones are always converted to their glycoconjugates by glycosyltransferases (GTs) for stability and further storage [12]. Therefore, the up-regulated expression of both EC:2.4.1.115 (UFGT) and EC:2.4.2.51 (UGT79B1) genes contributed to the glycosylation of the enhanced production of anthocyanidin and flavonol aglycones in the purple okra cultivars. Additionally, it is worth mentioning that the evident enhanced expression levels of some anthocyanin biosynthetic genes including EC:4.3.1.24 (PAL), EC:2.3.1.74 (CHS), and EC:2.4.1.115 (UFGT) in the pod skins of JO are still lower than those in BR and HY, according with the lowest content of anthocyanins in the four purple okra cultivars. In reference to the biosynthesis of flavonols, there were no significant differences in the expression levels of the key genes encoding flavonol synthase (FLS) among various okra cultivars. Overall, the up-regulated expression of anthocyanin biosynthetic genes accounts for the apparent anthocyanin accumulation in purple okra pods. Moreover, the enhanced metabolic flux towards anthocyanins in the flavonoid pathway augmented the production of flavonol glycosides with no changes in the mRNA levels of FLS.

### 3.6. Expression Analysis of Anthocyanin Biosynthesis Regulatory Proteins in Skins of the Four Okra Cultivars

**Figure 6:**
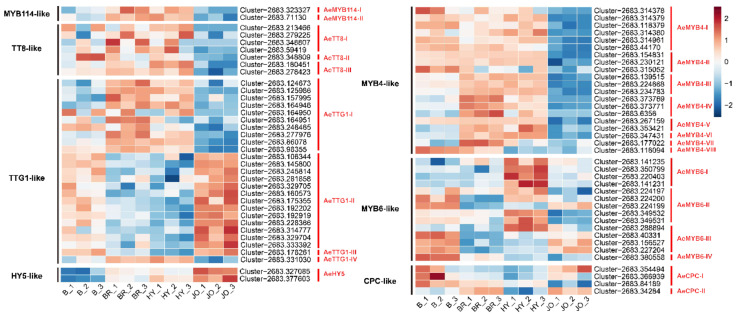
Heat map of the expression levels of the regulatory factors directly regulating anthocyanin biosynthesis in pod skins of the four okra cultivars. The FPKM values, log2-transformed, were used to generate the heat map. The blue color indicates low expression; red represents high abundance of expression.

To study the regulatory mechanisms underlying the significant upregulation of the anthocyanin biosynthetic genes in purple okra, the transcript levels of transcription factor encoding genes were further analyzed. It has been proved that the expression of most of the anthocyanin structural genes are directly activated by a ternary protein complex consisting of R2R3-MYB TF (AtPAP1, AtPAP2, AtMYB113, and AtMYB114), bHLH TF (AtTT8), and WD40 repeat protein (AtTTG1) in Arabidopsis [13]. In this study, two genes encoding R2R3 MYB proteins which show high homology with AtMYB114 were screened. As show in Figure 6, the expression of AeMYB114-I and AeMYB114-II was evidently up-regulated in the pod skins of BR and HY while they showed no increased transcripts in JO, in comparison with anthocyaninless cultivars (Burmese). Similarly, three genes encoding TT8-like proteins showed an increase in transcript abundance in the pod skins of BR and HY, but display no changes in JO when compared to B (Figure 6 and Table S8). Usually, the R2R3 MYB proteins of the MBW complex are thought to be the principal determinants of the target genes and confer anthocyanin production in different patterns or cell types [1]. Therefore, the regulatory mechanism of anthocyanin biosynthesis in JO might be probably different from that in BR and HY. The WDR proteins of the MBW complex were proved to serve a stabilizing function and interact directly with the bHLH proteins [1,36]. The genes encoding TTG1-like proteins show similar expression profiles between the pods of BR and HY, and different expression patterns in JO, further indicating the different regulatory mechanisms of anthocyanin biosynthesis among the purple okra cultivars (Figure 6 and Table S8). Based on the results mentioned above, the up-regulation of MYB114-like, TT8-like, and TTG1-like proteins should account for the abundant anthocyanin accumulation in the pod skins of BR and HY, but not JO.

In addition to the classical members of the MBW complex, transcription factors of WRKY, NAC, MADS box, and bZIP families have also been reported to play important regulatory roles in anthocyanin biosynthesis [21,22,23,37]. Most of these transcription factors have been proved to indirectly regulate the production of anthocyanins via MBW complex, while bZIP family transcription factor SlHY5 could directly recognize and bind to the promoters of anthocyanin biosynthetic genes, CHS and DFR, to trigger the accumulation of anthocyanins by inducing the transcriptional activation of target genes [38]. Our transcriptome data revealed that one differentially expressed HY5-like gene AeHY5 displayed significantly elevated expression levels in all purple okra cultivars in contrast to the green cultivar, with the highest transcript abundance in JO, indicating HY5-like protein might play vital regulatory roles in anthocyanin biosynthesis in purple okra, especially in JO. We further analyzed the transcripts of both AeCRY1 and AeCRY2 and they exhibited specifically increased mRNA levels in the pod skins of JO and decreased levels in both BR and HY when compared to B (Figure S3). Upon exposure to light, CRY proteins are activated, and these photoreceptors can bind to COP1, thereby releasing HY5 to regulate downstream anthocyanin biosynthetic genes primarily through directly binding to the promoter regions of target genes [38]. Hence, we also analyzed the expression levels of COP1-like genes and the results showed the expression profiles were more similar with that of AeCRY1 and AeCRY2 (Figure S3). Altogether, these results suggested HY5-like proteins might trigger anthocyanin biosynthesis in the pod skins of JO. Additionally, two NAC genes (AeANAC078-I and AeANAC078-II), with high homology to ANAC078 protein, were differentially expressed between the purple and green okra cultivars (Figure S3). AeANAC078-like genes were only up-regulated in the pods of BR, compared with B. Unexpectedly, the MADS genes, homologs of the bilberry VmTDR4 [21], and the WRKY genes, homologous to Arabidopsis TTG2 [22], showed no obvious changes (*p* < 0.01) in transcript levels (data not shown).

Except the activators, the R3-MYB and R2R3-MYB repressors for anthocyanin biosynthesis have also been characterized (Figure 6). Examples of R2R3-MYB repressors include FaMYB1, PhMYB27 and AtMYB4 [17,39,40] and these negative regulators, harboring the C-terminal domains (for example, the ethylene response factor-associated amphiphilic repression (EAR) motif), repress the transcription of anthocyanin biosynthetic genes [14]. R3-MYB repressors including AtMYBL2, AtCPC and PhMYBX act as competitive inhibitors by binding to bHLH proteins, resulting in disorders of the assemblage of a functional MBW complex [18,19,20,40]. In this study, eight genes encoding MYB4-like proteins were differentially expressed between the purple and green okra cultivars. Among them, the transcripts of the AeMYB4-III, AeMYB4-IV, AeMYB4-V, AeMYB4-VI and AeMYB4-VII were only up-regulated in BR and HY, in contrast with B (Figure 6). All the MYB4-like proteins display a significant decreased expression in pod skins of JO, implying the limited expression of MYB4-like repressors facilitated anthocyanin accumulation in JO. Intriguingly, most of the MYB6-like proteins which show high homology with AtMYBL2 exhibited a significant increase in gene expression levels specifically in HY, but BR and JO, suggesting the evident diversities of genetic backgrounds within the purple okra cultivars (Figure 6). In addition, two genes encoding CPC-like proteins (AeCPC-I and AeCPC-II) showed lower transcript abundance only in HY but no obvious changes in expression levels in other two purple okra cultivars when compared to B (Figure 6). These results suggested the expression profiles of the negative regulators for anthocyanin biosynthesis varied greatly among the purple okra cultivars.

**Figure 7:**
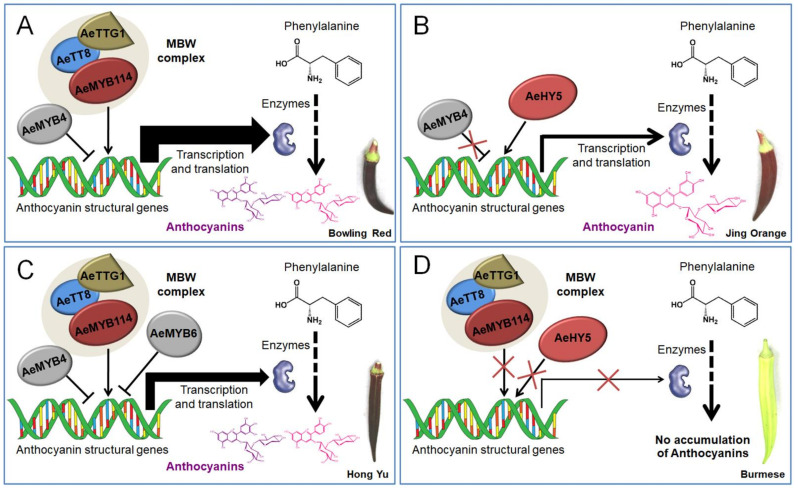
Proposed working models explaining the internal mechanisms for anthocyanin pigmentation in okra pods. Four putative working models underlying anthocyanin accumulation for Bowling Red (**A**), Jing Orange (**B**), Hong Yu (**C**), and Burmese (**D**) were provided respectively.

Therefore, four independent working models explaining the accumulation of anthocyanins and flavonols in the okra pods were proposed (Figure 7). The regulatory mechanisms underlying the accumulation of anthocyanins and flavonols varied depending on the individual okra cultivar. Overall, a classical MBW complex consisting of AeMYB114, AeTT8, and AeTTG1 along with AeMYB4 or AeMYB6 regulate the accumulation of anthocyanins via the transcriptional regulation of structural genes in BR and HY, while the HY5-like protein plays a crucial regulatory role in the accumulation of anthocyanins and flavonols by directly binding the promoters of anthocyanin structural genes in JO. Subsequently, the upregulated structural genes increased metabolic flux toward the accumulation of the anthocyanins and flavonols, contributing to the coloration of the purple okra pods (Figure 7A–C). Remarkably, the increased degrees of the expression levels of structural genes in BR, controlled by MBW complex and the repressor AeMYB4, was higher than that in HY, regulated by the MBW complex and two repressors including AeMYB4 and AeMYB6, and with the lowest increased sizes in JO, which further contributed to the highest anthocyanin content of pod skin in BR, followed by that in HY with the lowest anthocyanin content in JO (Table 1) and finally resulted in the coloration of the pods of Bowling Red, Hong Yu, and Jing Orange ranging from purplish to red (Figure 7A–C). Conversely, the low transcriptional activity of both the MBW complex and HY5-like protein failed to activate the transcription of structural genes and resulted in a defect in anthocyanin biosynthesis, which eventually leads to the formation of the green pod (Figure 7D). Although the expression profiles of the regulatory genes indicated that the molecular mechanisms might be more complicated than we speculated. However, the comparative transcriptome analyses of the regulatory genes associated with flavonoid pathway expands our understanding of the regulatory mechanisms of anthocyanin and flavonoid accumulation in okra.

## 4. Discussion

In this study, we found that the coloration of the purple okra pod mainly arises from the accumulation of both cyanidin-based and delphinidin-based anthocyanins in most of varieties (Hong Yu, Bowling Red, and Burgundy), except for Jing Orange. Generally, natural components of flavonoids identified in plants are mostly glycosylated and/or acetylated which are usually more stable than aglycone form in plant cells [1,14]. The main anthocyanins in purple okra pods are delphinidin 3-*O*-sambubioside and cyanidin 3-*O*-sambubioside. Both the two anthocyanins display a highly structural homology where the common building blocks are anthocyanidin chromophore, and sugars. The sambubiose moiety is bound at position 3 of anthocyanidin chromophore and the glycosylation is highly conserved between different components in all the purple cultivars, indicating a characteristic modification pattern in okra [36]. The key differentiation is anthocyanidin chromophore (delphinidin and cyanidin) where the hydroxylation occurs at position 3′ and 4′ of the B ring [30]. Comparative transcriptome analysis between three purple and one green okra cultivars reveals that the significant up-regulation of most of anthocyanin biosynthetic genes is responsible for the accumulation of anthocyanins in pod skins of BR, HY and JO, which is consistent with findings in other plant [5,28,29]. Simultaneously, it is proposed that the enhanced expression of biosynthetic genes raised the metabolic flux of flavonoid biosynthetic pathway, which leads to the elevated production of flavonol glycosides in the pod skins of BR, HY, and JO, compared with B. F3′5′H functions essentially in the sequentially production of dihydromyricetin, myricetin and delphinidin based flavonoids [29,33]. For instance, the lack of F3′5′H activity leads to the general inability of cruciferous plants to synthesize delphinidin-based anthocyanins [1,6]. Notably, the low mRNA levels of the F3′5′H gene in both Jing Orange and Burmese were responsible for the absent of delphinidin-based anthocyanins in Jing Orange and the lack of myricetin-based flavonol glycosides in both Burmese and Jing Orange.

Many studies have shown that anthocyanin structural genes are directly triggered by a ternary MBW protein complex consisting of R2R3-MYB, basic-helix-loop-helix (bHLH), and WD-repeat proteins widely in the plant kingdom [1,5,15,16]. Besides, partial members of R3-MYB and R2R3-MYB proteins negatively regulate of the accumulation of anthocyanins and flavonols [16,18]. Consequently, further studies of the differentially expressed transcription factors indicated that MYB114-like, TT8-like, and TTG1-like proteins along with AeMYB4 or AeMYB6 transcription factors coordinately regulate anthocyanin accumulation via activating the transcription of anthocyanin biosynthetic genes, concurrently resulting in the significant accumulation of flavonol glycosides as byproducts in pod skins of BR and HY, excluding JO. As for JO, HY5-like transcription factors were speculated to play a crucial regulation role in the accumulation of anthocyanins and flavonol glycosides [40]. In summary, four independent working models explaining the accumulation of anthocyanins and flavonol glycosides at both metabolic and molecular levels corresponding different purple cultivars were proposed in this study. Altogether, these results improve our understanding of the anthocyanin accumulation and the underlying molecular mechanisms in okra pod, and provide strong supports for the development of okra pod as a functional food in the future.

## Figures and Tables

**Table 1:** Anthocyanin levels (mg/g dry weight) in pod skins of the four purple cultivars (n = 3).

No. ^I^	RT ^II^ (min)	[M + H]^+^ (*m*/*z*)	MS/MS (*m*/*z*)	Compound	Hong Yu	Bowling Red	Burgundy	Jing Orange
1	4.01	597.1500	303.05	Delphinidin 3-*O*-sambubioside	3.47 ± 0.13 ^c^	7.00 ± 0.22 ^a^	4.29 ± 0.11 ^b^	Nd ^d^
2	4.97	581.1580	287.06	Cyanidin 3-*O*-sambubioside	3.52 ± 0.14 ^c^	4.05 ± 0.12 ^b^	3.06 ± 0.10 ^d^	4.41 ± 0.17 ^a^
Total					6.99 ± 0.16	11.05 ± 0.25	7.35 ± 0.16	4.41 ± 0.17

^I^ No corresponds to the elution order by UHPLC analysis in Figure 1. **^II^** RT, retention time. Nd, not detected. Different letters indicate a significant difference at *p* < 0.05.

**Table 2:** Flavonols identified (mg/g dry weight) in pod skins of 15 okra cultivars (n = 3).

No. ^a^	RT ^b^ (min)	λ Max: Band I, Band II (nm)	[M+H]^+^ (*m*/*z*)	MS/MS (*m*/*z*)	Compound
1	7.07	258, 354	613.1729	481.10/319.05	Myricetin 3-*O*-glucose-7-*O*-xyloside
2	8.03	258, 358	481.1045	319.05	Myricetin 3-*O*-glucoside
3	8.54	258, 354	597.0500	465.11/303.05	Quercetin 3-*O*-glucose-7-*O*-xyloside
4	10.02	258, 354	465.1090	303.05	Quercetin 3-*O*-glucoside
5	11.07	254, 354	611.1694	479.12/317.07	Isorhamnetin 3-*O*-glucose-7-*O*-xyloside
6	12.22	266, 358	449.1140	287.06	Kaempferol 3-*O*-glucoside

^a^ No corresponds to the elution order by UHPLC analysis in Figure 2. ^b^ RT, retention time.

**Table 3:** Flavonol levels (mg/g dry weight) in pods of 15 okra cultivars (n = 3).

Okra Cultivar	Flavonoid						Total
	Myricetin 3-*O*-Glucose-7-*O*-xyloside	Myricetin 3-*O*-Glucoside	Quercetin 3-*O*-Glucose-7-*O*-xyloside	Quercetin 3-*O*-Glucoside	Isorhamnetin 3-*O*-Glucose-7-*O*-xyloside	Kaempferol 3-*O*-Glucoside	
Hongyu	0.52 ± 0.01 ^b^	0.08 ± 0.01	3.72 ± 0.07 ^c^	0.34 ± 0.01 ^c^	0.14 ± 0.01 ^c^	0.05 ± 0.01	4.85 ± 0.02
Bowling Red	0.80 ± 0.02 ^a^	0.10 ± 0.01	4.07 ± 0.07 ^b^	0.32 ± 0.01 ^c^	0.13 ± 0.01 ^c^	0.05 ± 0.01	5.47 ± 0.03
Burgundy	0.56 ± 0.02 ^c^	0.07 ± 0.01	4.42 ± 0.11 ^a^	0.36 ± 0.01 ^b^	0.13 ± 0.01 ^c^	0.05 ± 0.01	5.59 ± 0.04
Jing Orange	Nd	Nd	4.55 ± 0.16 ^a^	0.39 ± 0.01 ^a^	0.10 ± 0.05 ^c^	0.04 ± 0.01	5.08 ± 0.08
Alabama Red	Nd	Nd	0.66 ± 0.03 ^e^	0.04 ± 0.01 ^e^	0.18 ± 0.02 ^b^	Nd	0.88 ± 0.02
Hill Country	Nd	Nd	0.74 ± 0.06 ^e^	0.05 ± 0.01 ^e^	0.23 ± 0.03 ^ab^	Nd	1.02 ± 0.03
Lvruyi	Nd	Nd	0.74 ± 0.05 ^e^	0.06 ± 0.01 ^e^	0.10 ± 0.01 ^d^	Nd	0.90 ± 0.02
Clemson Spineless	Nd	Nd	0.47 ± 0.02 ^e^	0.03 ± 0.01 ^ef^	0.19 ± 0.01 ^b^	Nd	0.69 ± 0.01
Eagle Pass	Nd	Nd	0.64 ± 0.02 ^e^	0.03 ± 0.01 ^ef^	0.20 ± 0.01 ^b^	Nd	0.87 ± 0.01
Gold Coast	Nd	Nd	0.37 ± 0.04 ^f^	0.02 ± 0.01 ^f^	0.14 ± 0.02 ^c^	Nd	0.53 ± 0.02
Star of David	Nd	Nd	0.91 ± 0.06 ^c^	0.09 ± 0.01 ^d^	0.19 ± 0.02 ^b^	Nd	1.19 ± 0.03
Stubby	Nd	Nd	0.99 ± 0.02 ^c^	0.10 ± 0.01 ^d^	0.26 ± 0.01 ^a^	Nd	1.35 ± 0.01
Burmese	Nd	Nd	0.29 ± 0.02 ^f^	Nd	0.08 ± 0.01 ^d^	Nd	0.37 ± 0.01
Emerald	Nd	Nd	0.37 ± 0.05 ^f^	0.01 ± 0.01 ^f^	0.16 ± 0.02 ^bc^	Nd	0.54 ± 0.02
Perkins’ Long Pod	Nd	Nd	0.94 ± 0.02 ^c^	0.08 ± 0.01 ^d^	0.12 ± 0.01 ^cd^	Nd	1.14 ± 0.02

Nd, not detected. Different letters indicate a significant difference at *p* < 0.05.

## Supplementary Materials

The following are available online at https://www.mdpi.com/article/10.3390/foods10092180/s1, Figure S1. The length distribution of the transcripts and unigenes assembled by the Trinity program in okra. Figure S2. Principal component analysis of the four okra samples based on the gene expression profiles. Figure S3. Heat map of the expression levels of the regulatory factors involved in anthocyanin biosynthesis in pod skins of the four okra cultivars. The FPKM values, log2-transformed, were used to generate the heat map. The blue color indicates low expression and the red represents high expression abundance. Figure S4. Phylogenetic analysis of MYB repressors from okra along with negative anthocyanin and flavonol regulators of MYB family characterized in other species (1000 bootstraps). Table S1. Summary of the illumina sequencing of okra. Table S2. A brief summary of the sequence assembly after illumina sequencing of okra. Table S3. Summary of the sequence annotation after illumina sequencing of okra. Table S4. Summary of the differential expression unigenes for B vs. BR, B vs. HY, and B vs. JO. Table S5. The functional annotation of the genes falling into subclass 2. Table S6. The functional annotation of the genes falling into subclass 6. Table S7. Summary of the core conserved differential expression unigenes across the three compared groups (B vs. BR, B vs. HY and B vs. JO groups). Table S8. Summary of the differential expression genes associated with potential regulation of anthocyanin and flavonol biosynthesis.
