# Supplementary figures and images for: Comparative Transcriptome Analysis of the Accumulation of Anthocyanins Revealed the Underlying Metabolic and Molecular Mechanisms of Purple Pod Coloration in Okra (Abelmoschus esculentus L.)
Source: Foods. 2021 Sep 14;10(9):2180. doi: 10.3390/foods10092180 (PMC8471371; doi:10.3390/foods10092180)

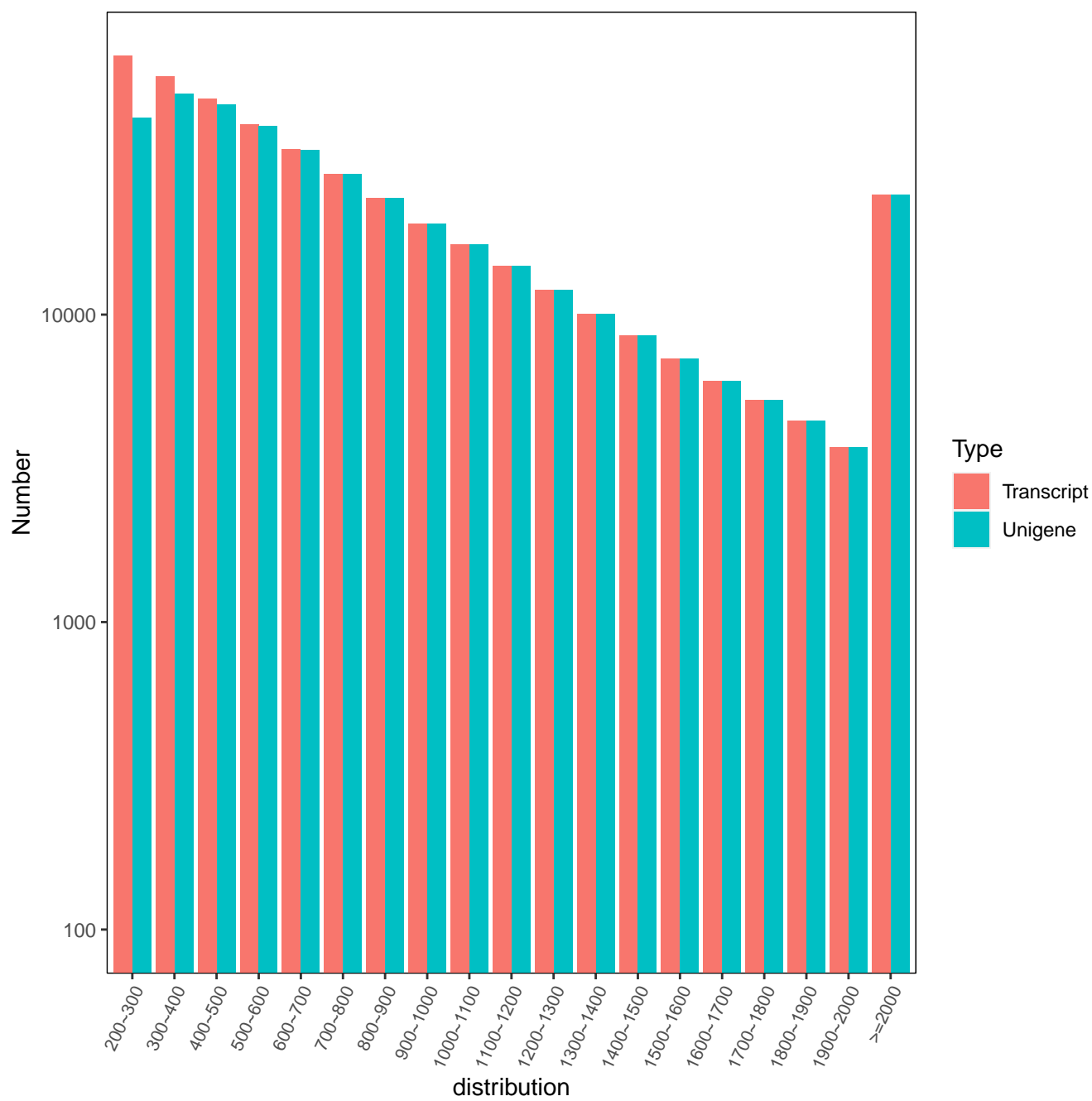

Supplement: Supplementary file 1 [file foods-10-02180-s001.zip › Supplementary Tables and Figures/Supplementary Figure 1.pdf]

2D PCA Plot

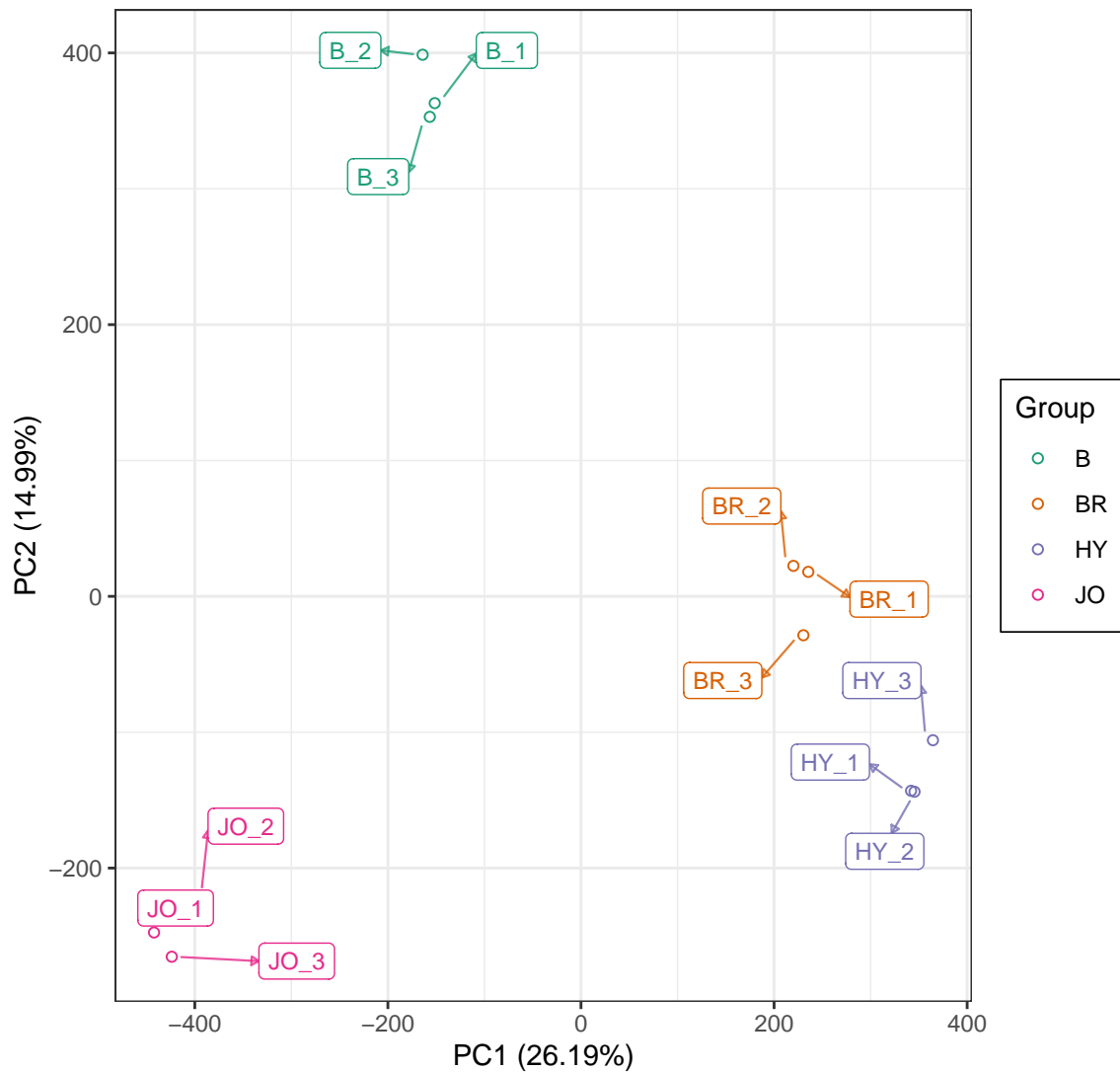

Supplement: Supplementary file 1 [file foods-10-02180-s001.zip › Supplementary Tables and Figures/Supplementary Figure 2.pdf]

ANAC078-like

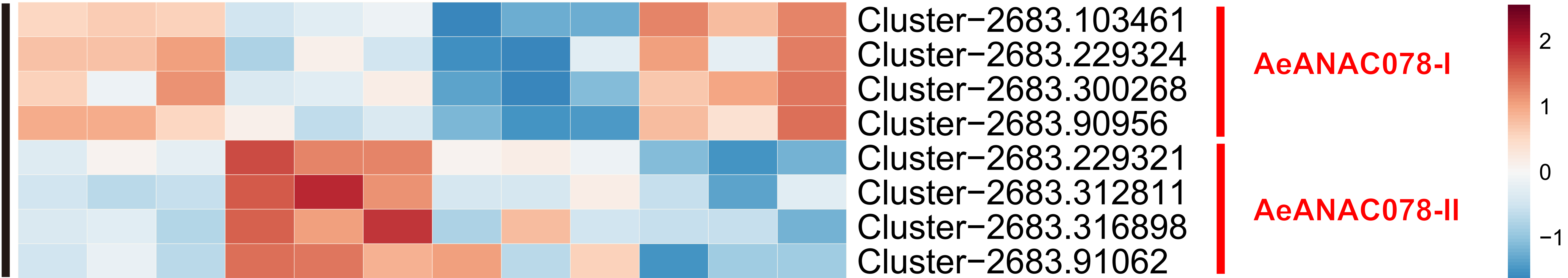

CRY1-like

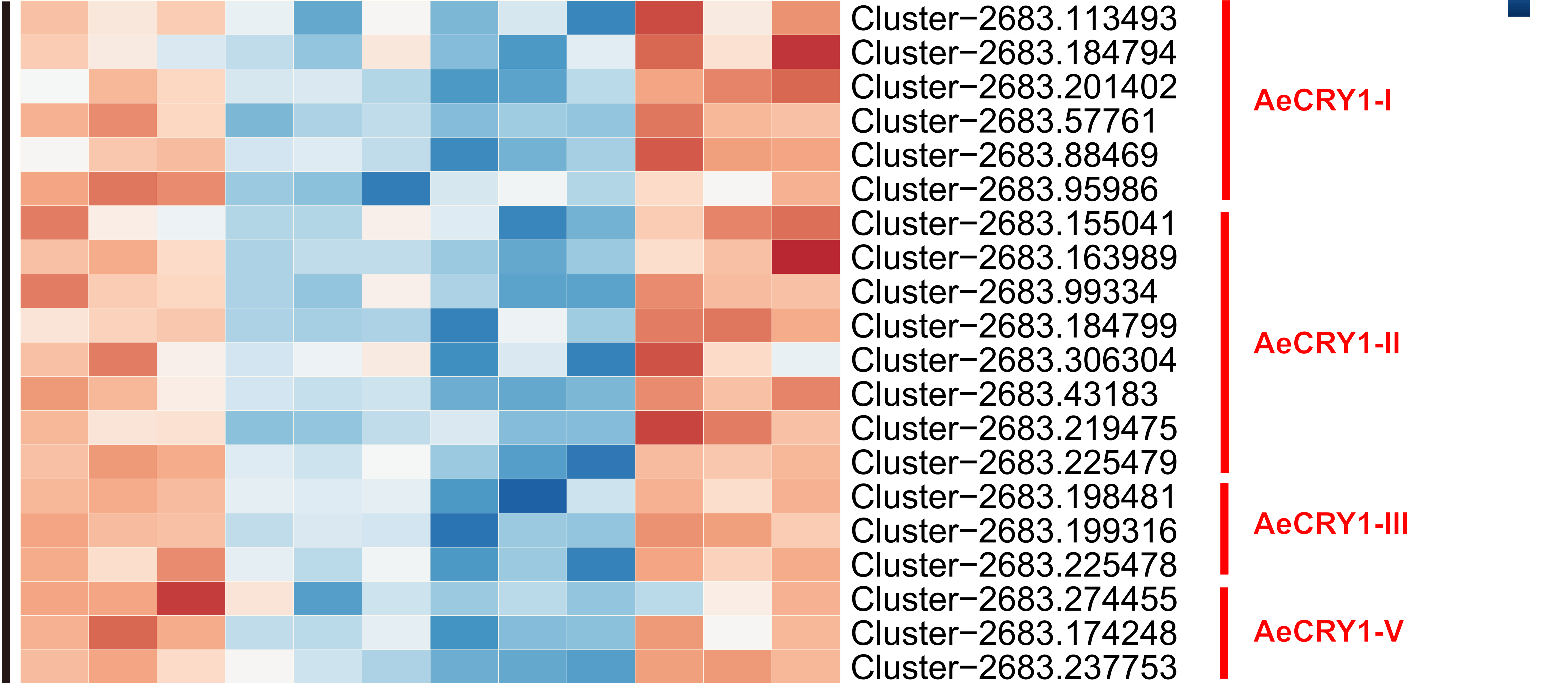

CRY2-like

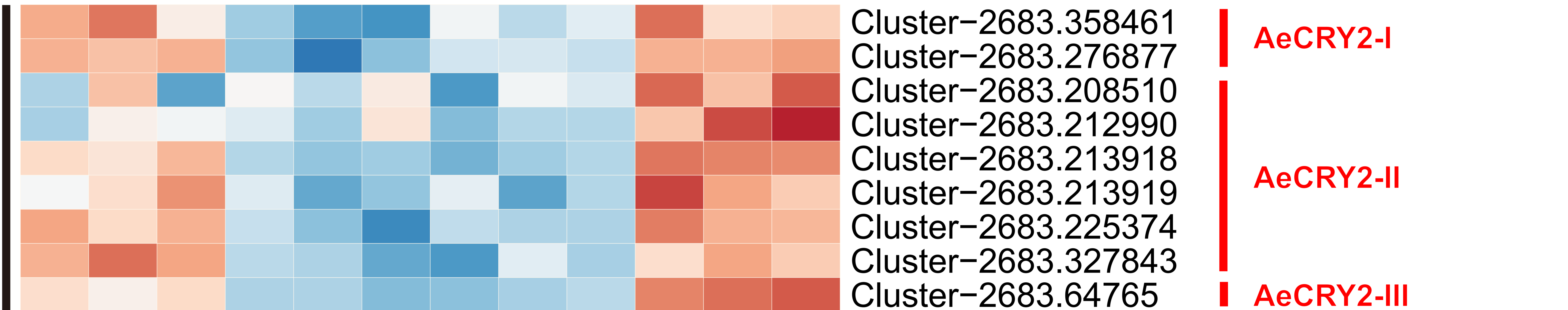

COP1-like

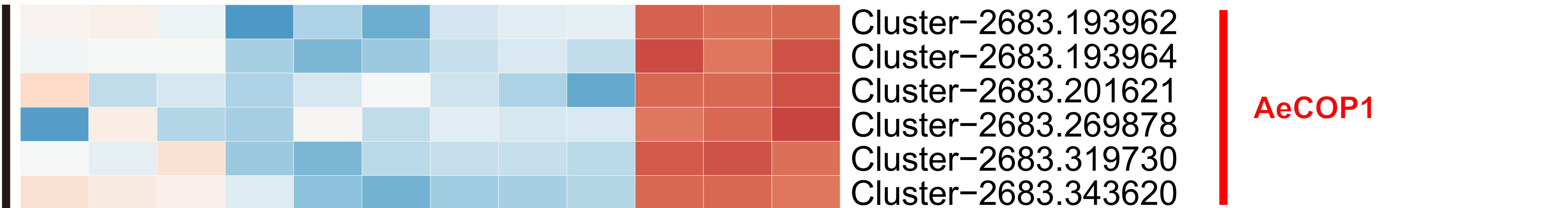

B\_1 B\_2 B\_3 BR\_1 BR\_2 BR\_3 HY\_1 HY\_2 HY\_3 JO\_1 JO\_2 JO\_3

Supplement: Supplementary file 1 [file foods-10-02180-s001.zip › Supplementary Tables and Figures/Supplementary Figure 3.pdf]

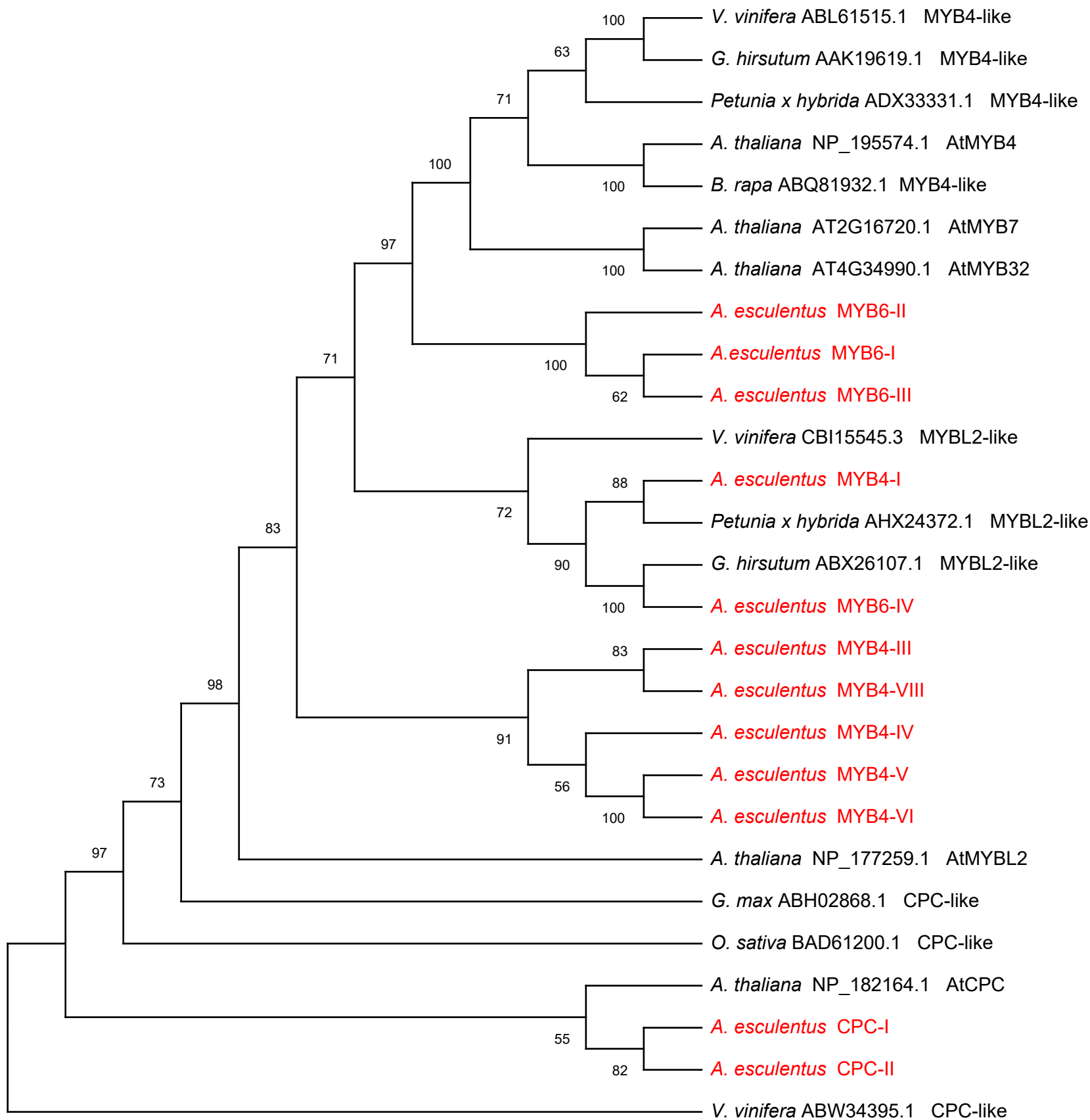

Supplement: Supplementary file 1 [file foods-10-02180-s001.zip › Supplementary Tables and Figures/Supplementary Figure 4.pdf]
